# Prevalence of *Borrelia miyamotoi* in *Ixodes* Ticks in Europe and the United States

**DOI:** 10.3201/eid2010.131583

**Published:** 2014-10

**Authors:** Chris D. Crowder, Heather E. Carolan, Megan A. Rounds, Vaclav Honig, Benedikt Mothes, Heike Haag, Oliver Nolte, Ben J. Luft, Libor Grubhoffer, David J. Ecker, Steven E. Schutzer, Mark W. Eshoo

**Affiliations:** Ibis Biosciences, Carlsbad, California, USA (C.D. Crowder, H.E. Carolan, M.A. Rounds, D.J. Ecker, M.W. Eshoo);; Biology Centre ASCR, Ceske Budejovice, Czech Republic (V. Honig, L. Grubhoffer);; Laboratory of Dr. Brunner, Constance, Germany (B. Mothes, H. Haag, O. Nolte);; University of Tübingen, Tübingen, Germany (B. Mothes); Zentrum für Labormedizin, St. Gallen, Switzerland (O. Nolte);; State University of New York at Stony Brook School of Medicine, Stony Brook, New York, USA (B.J. Luft);; Rutgers New Jersey Medical School, Newark, New Jersey, USA (S.E. Schutzer)

**Keywords:** Borrelia, Borrelia miyamotoi, relapsing fever, emerging pathogen, electrospray ionization mass spectrometry, PCR-ESI/MS, ticks, Ixodes, bacteria

## Abstract

*Borrelia miyamotoi*, a relapsing fever-related spirochete transmitted by *Ixodes* ticks, has been recently shown to be a human pathogen. To characterize the prevalence of this organism in questing *Ixodes* ticks, we tested 2,754 ticks for a variety of tickborne pathogens by PCR and electrospray-ionization mass spectrometry. Ticks were collected from California, New York, Connecticut, Pennsylvania, and Indiana in the United States and from Germany and the Czech Republic in Europe from 2008 through 2012. In addition, an isolate from Japan was characterized. We found 3 distinct genotypes, 1 for North America, 1 for Europe, and 1 for Japan. We found *B. miyamotoi* infection in ticks in 16 of the 26 sites surveyed, with infection prevalence as high as 15.4%. These results show the widespread distribution of the pathogen, indicating an exposure risk to humans in areas where *Ixodes* ticks reside.

## Abstract 2

Infection rates of *Ixodes* ticks with *Borrelia miyamotoi* in Europe and the United States vary greatly based upon location.

*Ixodes* ticks can transmit a variety of pathogens, including viruses, bacteria, and protozoa (*1*). *Borrelia* spirochetes are one of the genera of bacteria transmitted by *Ixodes* ticks. Most *Borrelia* that infect ticks belong to the *Borrelia burgdorferi* senso lato group and include *B. burgdorferi* senso stricto, *B. garinii*, and *B. afzelii*, all of which cause Lyme disease in humans (*1*). *Borrelia miyamotoi* has been found in a variety of *Ixodes* ticks and is more closely related to the relapsing fever spirochetes that infect soft ticks than to the bacteria that cause Lyme disease (*2*).

*B. miyamotoi* found in Europe and the United States also cause disease in humans (*3*–*5*). A study in Russia has shown that the spirochete *B. miyamotoi* has the ability to infect humans; infections with *B. miyamotoi* cause symptoms similar to those seen with relapsing fever, as well as erythema migrans-like skin lesions on rare occasions (*6*). *B. miyamotoi* has been found in ticks of the following species: *Ixodes scapularis* and *I. pacificus* in the United States, *I. persulcatus* in Japan, and *I. ricinus* and *I. persulcatus* in Europe and Asia (*2*,*7*–*11*). In North America, *B. miyamotoi* has been found as far north as the Canadian provinces of Ontario and Nova Scotia (*12*). In the United States, the geographic range of *B. miyamotoi* is from the Northeast to California and has been reported as far south as Tennessee (*7*,*8*,*13*–*15*). Previous studies have shown that *B. miyamotoi* can be placed into different genetic groups based upon its geographic location and has some variation within the genographic groups (*6*,*9*). 

To examine the prevalence distribution and diversity of *B. miyamotoi* in *Ixodes* ticks, we screened individual ticks by PCR and electrospray ionization mass spectrometry (PCR/ESI-MS) to detect tickborne pathogens, including *B. miyamotoi* (*16*). This approach has been used to characterize tickborne microorganisms, including *Ehrlichia* and *Borrelia*, from clinical specimens, heartworms in canine blood, and naturally occurring tick endosymbionts (*16*–*19*). Ticks that tested positive for *B. miyamotoi* were further characterized by using a *Borrelia* genotyping assay to assess genetic diversity (*20*).

## Materials and Methods

### *B. miyamotoi* Culture Isolate

The *B. miyamotoi* strain Fr74B was obtained by the Centers for Disease Control and Prevention (Fort Collins, CO, USA), as a culture isolate. This strain was originally isolated from an infected *Apodemus argenteus* field mouse from Japan. The DNA from this strain was isolated by diluting the culture 1:10 with phosphate-buffered saline and heating to 95°C for 10 min. The raw lysate was then used in the *Borrelia* PCR/ESI-MS genotyping assay (Abbott Laboratories, Des Plaines, IL, USA) at 1 μL per PCR well (*20*).

### *Ixodes* Tick Collection and Extractions

Ticks were obtained from most locations by flagging during 2008–2012. In Germany, a subset of ticks were also obtained after they were removed from persons. The species of *Ixodes* tick was determined by an entomologist and confirmed by the detection of the species-specific endosymbionts (*19*). The numbers and locations of the collection sites are described in Table 1.

**Table 1:** Prevelance of *Borrelia miyamotoi* in *Ixodes* ticks, Europe and the United States, 2008–2012*

Region/subregion	Species	Total no. ticks tested (nymphs; adults)	No. ticks positive for *B. miyamotoi *(% of total)
Czech Republic			
Zavadilka	*I. ricinus*	153 (153; 0)	4 (2.6)
Blatna	*I. ricinus*	100 (100; 0)	2 (2.0)
Dacice	*I. ricinus*	93 (93; 0)	3 (3.2)
Netolice	*I. ricinus*	89 (89; 0)	0 (0)
Germany			
Constance	*I. ricinus*	226 (0; 48)*	4 (1.8)
United States			
Connecticut			
Fairfield County	*I. scapularis*	322 (309; 13)	16 (5.0)
Litchfield County	*I. scapularis*	18 (18; 0)	0
New London County	*I. scapularis*	29 (29; 0)	0
New York			
Dutchess County	*I. scapularis*	357 (357; 0)	2 (0.56)
Suffolk County	*I. scapularis*	180 (24; 156)	2 (1.1)
Westchester County	*I. scapularis*	44 (0; 44)	3 (6.8)
Pennsylvania			
Chester County	*I. scapularis*	80 (79; 1)	2 (2.5)
Indiana			
Pulaski County	*I. scapularis*	81 (0; 81)	10 (12.3)
California			
Alameda County	*I. pacificus*	22 (0; 22)	1 (4.5)
Del Norte County	*I. pacificus*	33 (0; 33)	0
Glenn County	*I. pacificus*	44 (0; 44)	0
Humbolt County	*I. pacificus*	74 (0; 74)	0
Lake County	*I. pacificus*	129 (0; 129)	0
Marin County	*I. pacificus*	85 (0; 85)	1 (1.2)
Mendocino County	*I. pacificus*	57 (0; 57)	2 (3.5)
Napa County	*I. pacificus*	65 (0; 65)	10 (15.4)
Orange County	*I. pacificus*	15 (0; 15)	0
Placer County	*I. pacificus*	250 (0; 250)	4 (1.6)
San Bernardino County	*I. pacificus*	18 (0; 18)	0
Santa Cruz County	*I. pacificus*	64 (0; 64)	0
Sonoma County	*I. pacificus*	126 (126; 0)	2 (1.6)

*A total of 119 ticks were removed from humans, and the life stage of 178 of the 226 ticks tested was not recorded.

Nucleic acids were extracted from ticks according to a published protocol by using bead-beating homogenization followed by isolation of RNA and DNA with DNeasy Blood and Tissue Kit columns (QIAGEN, Valencia, CA, USA) instead of the published QiaAmp Virus Elute Kits (*21*). A negative control consisting of a lysis buffer without a tick was with each set of extractions. Ticks from the United States were processed at Ibis Biosciences (Carlsbad, CA, USA). Ticks collected from the European countries were isolated at their respective sources. Nucleic acid samples from Germany and the Czech Republic were shipped to Ibis at ambient temperatures; those from Czech Republic were shipped after being stabilized by RNAstable (Biomatrica, San Diego, CA, USA) per the manufacturer’s instructions.

### Molecular Detection and Genotyping of *B. miyamotoi* from Nucleic Acid Extracts

*B. miyamotoi* was detected and identified by using a previously described broad-range PCR/ESI-MS assay designed to detect tickborne pathogens (*16*). For each set of samples analyzed with the assay, an extraction negative control sample as well as a PCR plate negative-control sample of water was included. A PCR-positive control was already built into the plate for each well in the form of a calibrant (*20*). Amplicons were analyzed by using a research use only PLEX-ID system (Abbott Laboratories). Samples positive for *B. miyamotoi* were further characterized by using a *Borrelia* PCR/ESI-MS genotyping assay as described that is designed to differentiate between *Borrelia* species and genotypes (*20*). PCR/ESI-MS assay provides genetic information about the PCR amplicon in the form of A, G, C, and T basecounts, and *B. miyamotoi* detection was defined as positive when one or more primer pairs produced an amplicon basecount signature that was unique to *B. miyamotoi*. Although most researchers agree that the nymphal stage of *Ixodes* ticks is the most epidemiologically essential life stage for transmission of *B. burgdorferi* sensu lato, because little is known about the transmission of *B. miyamotoi* from *Ixodes* ticks to humans, the data for both nymphs and adults were combined.

### Sequence Confirmation of *B. miyamotoi* Detections

Representative samples positive for *B. miyamotoi* were selected for 16S Sanger sequencing. Primers were designed to amplify a 676-bp region of the 16S rRNA gene for *Borrelia*. A M13 tag was added to each primer for sequencing. The M13 forward sequence tag was 5′-CCC AGT CAC GAC GTT GTA AAA CG-3′, and the reverse tag was 5′-AGC GGA TAA CAA TTT CAC ACA GG-3′. The forward primer used was 5′-M13-CGC TGG CAG TGC GTC TTA AG-3′, and the reverse primer was 5′-M13-GCG TCA GTC TTG ACC CAG AAG TTC-3′. The amplification of the 16S rRNA genes was performed in a 50 μL reaction containing 1 μL nucleic acid extract, 1 unit of Platinum Taq High Fidelity polymerase (Invitrogen, Carlsbad, CA, USA) or Immolase Taq (Bioline, Randolph, MA, USA), the manufacturer’s PCR buffer, 2.0 mmol/L MgSO_4_, 200 μmol/L dATP, 200 μmol/L dCTP, 200 μmol/L dTTP, 200 μmol/L dGTP (Bioline), and 250 nmol/L of each primer. The following PCR cycling conditions were used on an MJ Dyad 96-well thermocycler (Bio-Rad Inc., Hercules, CA, USA): 95°C for 2 min, followed by 8 cycles of 95°C for 15 s, 50°C for 45 s, and 68°C for 90 s, with the 50°C annealing temperature increasing 0.6°C for each cycle. PCR was continued for 37 additional cycles of 95°C for 15 s, 60°C for 15 s, and 68°C for 60 s. The PCR cycle ended with a final extension of 4 min at 72°C. Reactions were visualized by electrophoresis on 1% agarose gels to ensure the presence of appropriately-sized products before being sent to SeqWright (Houston, TX, USA) for purification and sequencing with M13 primers. Resulting sequences were trimmed of primer sequences and a consensus created. The consensus sequence was analyzed with NCBI BLAST (http://blast.ncbi.nlm.nih.gov/Blast.cgi) against the nucleotide database to determine the species.

## Results

### Multilocus PCR/ESI-MS Genotyping of *B. miyamotoi*

The multilocus *Borrelia* PCR/ESI-MS genotyping assay differentiates strains and species of *Borrelia* by their unique combination of basecount signatures. To characterize the prevalence of *B. miyamotoi* in *Ixodes* ticks we examined the basecount signatures from ticks that were positive for *B. miyamotoi*. Positive specimens from each of the 3 regions (United States, Europe, and Japan) typically produced basecount signatures at 5 of the 8 loci evaluated in the *Borrelia* genotyping assay. Based upon these 5 signatures, *B. miyamotoi* from the United States, Europe, and Japan are distinct genotypes (Table 2). All the specimens from North America had the same basecount signatures for the 5 detecting primer pairs. A separate signature combination was found for all of the European isolates detected in ticks from Germany and the Czech Republic. A third signature was observed from the CDC culture isolate from the Japanese strain. Although all 3 genotypes shared the same basecount for the locus BCT3515, the European genotype did not have any other basecount signatures in common with the other 2 genotypes. The North American and Japanese genotypes had the same signatures for 2 of the 4 remaining loci, BCT 3519 and BCT3511. We detected *B. miyamotoi* with 3 or more primers in the *Borrelia* genotyping assay in all but 4 of the 68 positive specimens. Several factors may explain why all 5 primers did not detect the bacteria, including nucleic acid quality and quantity or differences in primer sensitivities.

**Table 2:** *Borrelia miyamotoi* PCR/ESI-MS basecount signatures*

Region	Genotype	BCT3515 (*rplB*)	BCT3517 (*flaB*)	BCT3519 (*hbb*)	BCT3520 (*hbb*)	BCT3511 (*gyrB*)
Europe	1	A13G22C15T18	A41G30C23T27	A41G29C19T46	A52G29C13T47	A36G32C13T35
North America	2	A13G22C15T18	A43G28C23T27	A40G30C18T47	A52G30C13T46	A37G31C13T35
Japan	3	A13G22C15T18	A41G29C23T28	A40G30C18T47	A53G29C13T46	A37G31C13T35

*PCR/ESI-MS, PCR and electrospray ionization mass spectrometry.

### Prevalence of *B. miyamotoi* in Europe and the United States

*I. ricinus* ticks from the Czech Republic and Germany in Europe and *I. scapularis* and *I. pacificus* ticks from 5 states in the United States were screened for *B. miyamotoi* by PCR/ESI-MS. *B. miyamotoi* was found in all regions examined in varying degrees (Table 1) and in all 3 *Ixodes* species examined. Germany had a low incidence rate; only 4 of the 226 ticks tested were infected (1.8%). Incidence of *B. miyamotoi* infection of ticks from the Czech Republic varied by region and ranged from 0% to 3.2% with an average infection rate of 2%. In North America, the infection rates of ticks varied from 0% to 15.4%. All negative controls were negative and all positive controls were positive.

### Sequence Confirmation of *B. miyamotoi* detections

Representative samples were selected for 16S rRNA sequencing: 1 sample from Pennsylvania in the United States, 1 from Germany, and 1 from the Czech Republic. The samples from Germany and the Czech Republic were identical (KF740842 and KF740841, respectively) and matched 99.11% (669 bp out of 675 bp) of the *B. miyamotoi* LB-2001 sequence, a North American isolate from the East Coast (GenBank accession no. NC_022079). The sample from Pennsylvania (KF740843) was identical (675 bp of 675 bp) to the *B. miyamotoi* LB-2001 sequence.

## Discussion

In this study, we identified 3 distinct *B. miyamotoi* genotypes in the United States, Europe, and Japan. Results show that *B. miyamotoi* is widely distributed across North America and Europe.We observed no genotypic differences using this PCR/ESI-MS assay between the *B. miyamotoi* detected in *I. scapularis* from the eastern US states and the midwest or between these bacteria and the *B. miyamotoi* detected in *I. pacificus* from California. In a study by Mun et al., a 766-bp region of the flagellin gene sequence were shown to have and a 0.9% difference between *B. miyamotoi* found in *I. pacificus* and those found in *I. scapularis* in the United States (*8*). However, our flagellin primers targeted a region of the flagellin gene that does not contain the differences identified by Mun et al., thus explaining why we found a single North American genotype. Previous studies that examined the sequence of the 16S rRNA gene from multiple *B. miyamotoi* strains indicated that strains from the United States and Europe were located in their own clusters (*6*). The Japanese strain FR64b grouped with isolates found in infected humans and *I. persulcatus* ticks in Russia, whereas the *B. miyamotoi* found in *I. ricinus* ticks from Russia grouped with those found in Europe (*6*). In our genetic analysis, the Japanese strain also differed from that found in *I. ricinus* in Europe.

Our study demonstrates that the presence of *B. miyamotoi* in *Ixodes* ticks is widespread across the regions examined and was observed in all 3 species of field-collected *Ixodes* ticks. In Europe we observed *B. miyamotoi* in ≈2.0% of *I. ricinus* ticks tested, consistent with the detection rates in other studies examining *I. ricinus* prevalence at other locations in Europe (*9*,*10*). Our detection rates were also similar to those seen in an earlier study on ticks from Mendocino County, California (*8*). *I. scapularis* ticks from the East Coast region (New York, Connecticut, and Pennsylvania) were found to have infection rates ranging from 0% to 6.8% for ticks. In Indiana, however, a much higher percentage, ≈12%, of *I. scapularis* ticks examined were infected with *B. miyamotoi*. Other studies have also shown that local site-to-site prevalence of *B. miyamotoi* can vary greatly from the overall regional mean (*13*).

Our study indicates that *B. miyamotoi* is likely present in any region where *Ixodes* ticks reside but that infection rates can vary greatly by region. Since the original description of *B. miyamotoi* as a human pathogen, studies have shown clinical infection in both healthy and immunocompromised patients in both Europe and the United States (*3*–*6*,*22*). If physicians know the regional infection rate in ticks, they will be alert for possible exposure risks for their patients. Standard Lyme borreliosis serologic tests offered by commercial laboratories cannot be relied on to detect *B. miyamotoi* infection in patients. *B. miyamotoi* has been shown to have transovarial transmission, suggesting that larval ticks may also pose a risk (*7*). Little is yet known about the transmission rates to humans, and further studies are required to better gauge the risk to humans in these *B. miyamotoi*-endemic regions.
